# Quality of life, body image and self-esteem in patients with unilateral transtibial amputations

**DOI:** 10.1038/s41598-021-91954-1

**Published:** 2021-06-15

**Authors:** Nuria Sarroca, José Valero, Javier Deus, Josefa Casanova, María José Luesma, Manuel Lahoz

**Affiliations:** 1 Zaragoza, Spain; 2 Department of Surgery, University Hospital, 50009 Zaragoza, Spain; 3 Checa Health Centre, Guadalajara, Spain; 4 Department of Human Anatomy and Histology, University of Zaragoza, Calle Domingo Miral s/n, 50009 Zaragoza, Spain

**Keywords:** Psychology, Rheumatology

## Abstract

Amputation represents a drastic impact on the patient’s body and perception. This cross-sectional study aims to analyse the aesthetic concern represented by body image, self-esteem and quality of life in patients with unilateral transtibial amputations of lower limbs compared to control group. People living with amputations present lower average levels than controls in all subscales of the SF-36 (Short Form 36 Health Survey) quality of life questionnaire, and in both the physical component summary and the mental component summary, although the difference is not statistically significant in the latter. These patients present a significantly lower mean score (*p* = 0.002) in the MBSRQ (Body-Self Relations Questionnaire) body image questionnaire: 2.64 ± 0.49 opposed to 3.16 ± 0.55 in controls. People living with amputations had a lower mean score on the Rosenberg Self-Esteem scale than controls (34.44 ± 4.61 v 36.04 ± 3.63). The results also show that amputation has a significant influence on the BI (Body image) of patients with unilateral transtibial amputations. SE (Self-Esteem) seems to be an aspect that is not significantly affected by lower limb amputation, although these patients scored a lower mean on the RSE scale compared to the control group. We consider it is highly relevant to assess QoL (Quality of life), BI and SE in patients after a lower limb amputation process.

## Introduction

The World Health Organisation (cited by Gallagher and Maclachlan, 2004) defines health as the “state of complete physical, mental and social well-being and not merely the absence of disease or infirmity”^1^.

Limb amputation is often an unavoidable procedure in the advanced condition of several diseases, such as diabetes mellitus (DM), peripheral arterial occlusive disease (PAOD), cancer disorders, trauma or infection, posing a dramatic impact on a patient’s life.

Amputation incidence ranges from 1.2 to 4.4 per 10,000 inhabitants in different countries^2,3^, and the majority (up to 90%) affect the lower limbs. It is estimated that these figures could double by 2050^3^.

An amputation leads to several limitations in the performance of social, professional and leisure activities^4,5^.

The World Health Organisation defines quality of life as “an individual's perception of his/her position in circumstances of the culture and values in which he or she lives and with respect to his/her goals, expectations, principles and concerns”^6^. Demographic and health factors have proven to be influential on quality of life^7^, including many facets of human life, such as physical, mental, spiritual and social aspects^8,9^.

In some cases, amputation may improve the patient's quality of life and daily functioning because chronic and progressive lower limb ischemia or chronic limb infection often cause severe pain, movement restriction, and disabilities in everyday tasks. In such cases, amputation is a procedure that actually decreases the disability level^8,10^, However, the fact remains that the integrity of the human body is disturbed, entailing diminished quality of life (QoL), along with reduced mobility, pain and physical integrity^4^.

Patients are affected psychologically and socially^4^. Psychological problems range from depression, anxiety and suicide in severe cases^4,11^.

It is obvious that the amputation of a lower limb, whatever its cause, has a strong repercussion on physical, functional and emotional aspects, affecting the QoL of the person living with the amputation^12–15^.

QoL is recognised as a major predictor of rehabilitation programmes and has mainly been used to evaluate the effectiveness of these programmes, or to compare people with amputations with diseased or normal population^16,17^. There is extensive literature on the different factors that influence QoL in people living with amputations. There is some confusion between these factors, which in turn depend on other sub-factors. This has led to discrepancies among authors in terms of how the patient's age, sex, type and level of amputation, time since the procedure, etc. influence the QoL of people living with amputations. In this sense, we find some authors who defend that these factors influence QoL^16,18–21^ and others who defend that factors such as age do not affect overall QoL^22^. There seems to be agreement that the factors that most determine a decrease in QoL of these patients are stump pain and phantom pain, together with walking distance, which is reduced due to the latter^18^. The presence of concomitant diseases negatively influences the physical and mental aspects of QoL^16^. However, the studies show the relevance, in terms of QoL, of patients using a prosthesis since this allows them to undertake more activities than those who do not use an artificial limb^21^, as well as the importance of patients’ greater job satisfaction^23^.

What is clear is that, after amputation, it is crucial to start active rehabilitation. This includes physical therapy and occupational therapy, encouraging the patient to use a prosthesis and return to his/her routine social activities^8,24^.

The two main pillars of the perception of one’s appearance are body image (BI) and self-esteem (SE)^25^.

BI is a person’s individual perception of his/her own body, and it is a dynamic and multidimensional process. This perception is affected both by internal factors such as age, gender, physical condition, as well as by external factors, which include social or environmental factors^26^.

SE is a positive or negative attitude towards oneself: an overall assessment of one’s self-worth or value^27,28^. SE encompasses beliefs and emotions, such a triumph, despair, pride and shame^29^.

The main aim of our study was to analyse QoL, SE and BI in patients with prosthetic unilateral transtibial amputations in relation to the general population.

## Materials and methods

### Study population

A cross-sectional study with a comparison group was performed on 25 patients with prosthetic unilateral transtibial amputations. Patients’ ages ranged between 18 and 70. They had no cognitive limitations to answer the questionnaires, and consented to participate in the study. Furthermore, at least one year had passed since the amputation to guarantee the patients’ ability to successfully ambulate with their prosthesis. Patients were recruited from databases of Chiropody Clinics and specialised Orthopaedics Centres during the period 2015–2017. A control group of another 25 people was also included for comparison purposes. The control group comprised healthy individuals, aged between 18 and 70, with no apparent osteoarticular or neurological disorders, and they came from Clínica Sarroca. Controls were paired with the cases by gender and age (± 5 years).

### Measurement instruments

A 91-item self-administered questionnaire, including QoL, BI and SE scales, was presented to each participant. In this study, the SF-36 Questionnaire was included as one of the health-related quality of life (HRQoL) instruments.

Further, the two main components of aesthetic perception in lower limb amputees were assessed using the Multidimensional Body Self Relations Questionnaire (MBSRQ) and the Rosenberg Self-Esteem Scale (RSE) Questionnaire.

### Quality of life: the short form (36) health survey (SF-36)

The SF-36 questionnaire is extensively used to assess generic health domains that are not specific to age, disease or treatment group. The questionnaire assesses the individual’s perception of their situation in life within the cultural and value context where they live, related to their goals, expectations, values and interests, as well as the impact on their health status^30,31^. It contains 36 items covering eight health-related quality of life (HRQoL) domains: bodily pain (BP), physical functioning (PF), role limitation due to physical problems (PR), role limitation due to emotional problems (ER), Vitality (V), social functioning (SF), mental health (MH), and general health perceptions (GH). The questions in each domain were written down, coded, summed, transformed and presented as a final score of 0 (worst quality of life) up to 100 (best quality of life). A physical component summary score (PCS) and a mental component summary score (MCS) can be calculated using the standardised score system^32^.

### Body image: body-self relations questionnaire (MBSRQ®)

The Body-Self Relations Questionnaire (MBSRQ®) is a 45-item questionnaire^33^ from which four factors emerge: (1) subjective importance of corporality (SIC; (2) physical-oriented behaviours (FOB); (3) self-assessed physical attractiveness (SAPA); and (4) caring for one’s external appearance (COEA). This permits distinguishing subjects on each one of the scales according to their score, and in general (the higher the score, the better the body image).

### Self-esteem: Rosenberg self-esteem questionnaire (RSE)

The Rosenberg Self-Esteem Scale (RSE) is a 10-item self-report questionnaire that assesses overall personal self-esteem on a summary scale^34^. Each item was rated on a four-point Likert scale from 1 (strongly agree) to 4 (strongly disagree), producing a cumulative score of 0 to 30, where high mean scores (computed) indicate high self-esteem^35^.

### Statistical analysis

Firstly, a descriptive analysis of the study variables was conducted. Relative and absolute frequencies were provided for categorical variables, and means and standard deviations (SD) were provided for quantitative variables. The relationship between categorical variables was assessed by means of the Chi-Squared test. The Mann–Whitney U test or Student’s T test were used to compare differences between two independent groups when the dependent variable was quantitative, according to normality criteria. The Pearson or Spearman correlation coefficient was used to study the linear relationship between two quantitative variables. The Komolgorov-Smirnov test was used to analyse the normality of variables.

We used a multivariate analysis of covariance (MANCOVA) to compare SF-36 and MBSRQ between both groups, since the outcome measures consisted of two related dependent variables.

The level of statistical significance was established for a *p* value of less than 0.05. The statistic program SPSS 22.0 for Windows (SPSS Ibérica, Madrid, Spain) was used throughout the entire analysis.

### Sample size calculation

We calculated the sample size for the main objective of the study based on the results of a pilot study carried out with the first 10 patients and controls. A sample size of 22 patients and 22 controls would be necessary to detect a difference of 4.6 or greater for the SF-36 Physical summary score, with 95% confidence and 80% power. Sample size calculation for two independent means of EPIDAT 4.2 was applied.

### Ethical considerations

The study was verified and approved by the Research Ethics Committee of the Community of Aragon (CEICA) (Registration nº: PI18/403), and it was conducted in agreement with the Helsinki Declaration (World Medical Association, 1964)^36^. All subjects (controls and people with amputations) signed an informed consent form prior to participating in the study.

## Results

Each group included 84% men (21/25) and 16% women (4/25). The average age of patients was 44.04 ± 12.92 years while that of controls was 38.44 ± 12.42 years. This difference between groups is not statistically significant (*p* = 0.125).

### Quality of life

#### SF-36 questionnaire

**Figure 1:**
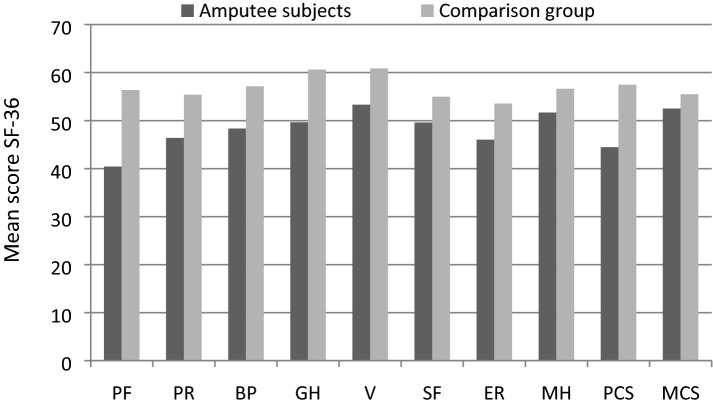
Questionnaire SF36 (mean ± SD).

People with amputations have lower mean levels than controls in all subscales of the SF-36 quality of life questionnaire, and in both the physical component summary and the mental component summary, although the difference is not statistically significant in the latter (Table 1). Scores are graphically represented in Fig. 1. We conducted a multivariate analysis of variance (MANCOVA), which confirms significant differences in the SF-36 PCS and MCS scores between groups, after adjusting for gender and age (Wilk´s Lambda *p* < 0.001). This analysis also confirms the results for the two independent scores (PCS *p* < 0.001, MCS *p* = 0.139).

**Table 1:** Data associated with Questionnaire SF36 (mean ± SD).

Scale	Amputee subjects	Comparison group	*p* value
Physical functioning	40.44 ± 8.19	56.36 ± 1.80	< 0.001*
Physical role	46.40 ± 9.10	55.40 ± 3.70	< 0.001*
Bodily pain	48.36 ± 10.02	57.16 ± 7.98	0.001*
General health	49.64 ± 8.87	60.64 ± 4.83	< 0.001**
Vitality	53.32 ± 6.60	60.88 ± 6.90	< 0.001**
Social functioning	49.60 ± 8.91	55.00 ± 4.33	0.008*
Emotional role	46.04 ± 10.40	53.56 ± 4.27	0.004*
Mental health	51.68 ± 8.18	56.64 ± 6.98	0.025**
Physical component	44.48 ± 7.84	57.48 ± 3.48	< 0.001**
Mental component	52.52 ± 7.74	55.52 ± 6.16	0.136**

*Mann–Whitney U Test.

**Student’s T Test.

### Body image

#### MBSRQ questionnaire

**Figure 2:**
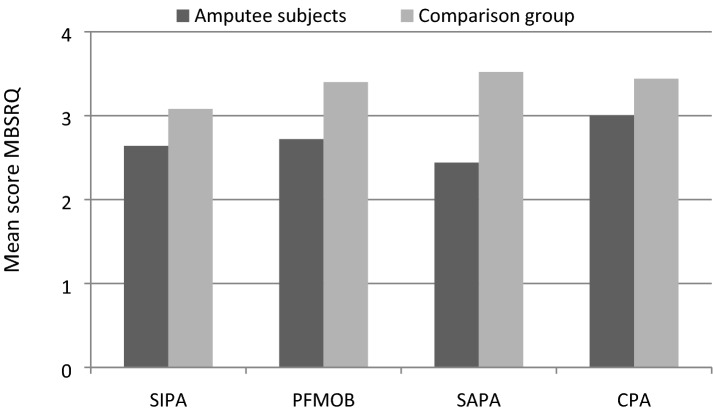
MBSRQ questionnaire (mean ± SD).

People with amputations have a significantly lower mean score (*p* = 0.002) on the MBSRQ body image questionnaire: 2.64 ± 0.49 opposed to 3.16 ± 0.55 in controls. In terms of subscales, people with amputations also have lower mean levels in subjective importance of corporality (SIC), fitness-oriented behaviours (FOB), Self-Assessed Physical Attractiveness (SAPA), and Caring for one’s External Appearance (COEA), as shown in Table 2. The scores are graphically represented in Fig. 2. MANCOVA analysis confirmed significant differences in the MBSRQ subscales between both groups after adjusting for gender and age (Wilk´s Lambda *p* = 0.004), also confirming the results for the independent subscales (SIPA *p* = 0.013, PFMOB *p* = 0.025, SAPA *p* = 0.001, CPA *p* = 0.108).

**Table 2:** Body image questionnaire MBSRQ subscales (mean ± SD).

Scale	Amputee subjects	Comparison group	*p* value*
Physical functioning	2.64 ± 0.49	3.08 ± 0.57	**0.008**
Physical role	2.72 ± 0.89	3.40 ± 0.82	**0.012**
Bodily pain	2.44 ± 0.92	3.52 ± 0.96	< **0.001**
General health	3.00 ± 0.87	3.44 ± 0.71	0.050

Statistically significant values *p* < 0.05 are marked in bold.

*Mann–Whitney U test.

### Self-esteem

#### Rosenberg questionnaire

People with amputations had a lower mean score on the Rosenberg Self-Esteem scale compared to controls (34.44 ± 4.61 v 36.04 ± 3.63). However, there were no statistically significant differences between the groups (*p* = 0.179).

88% of people with amputations (22/25) and 96% of controls (24/25) had high SE (Rosenberg of 30 to 40 points) with no statistically significant difference between groups (*p* = 0.609).

**Figure 3:**
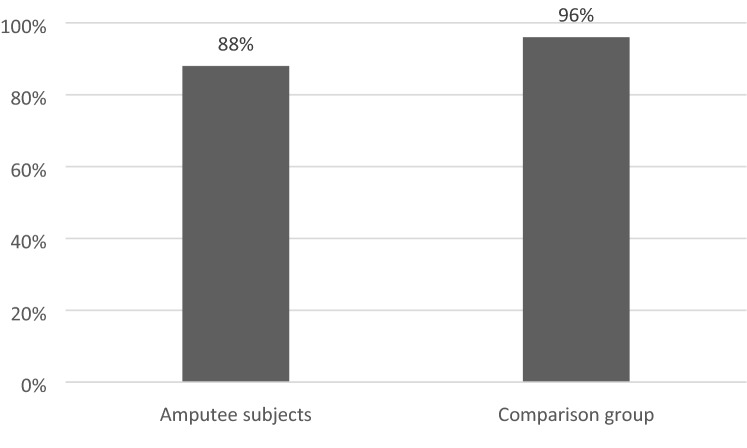
Participants with high self-esteem according to the Rosenberg Self-Esteem scale (*p* = 0.609).

The scores of the Rosenberg questionnaire are graphically represented in Fig. 3.

### Relationship between QoL, BI and SE

A statistically significant positive correlation (low or moderate) was observed between the three scores of QoL, BI and SE in study participants (Table 3).

**Table 3:** Relationship between the three questionnaires.

	Rosenberg	MBSRQ	PCS SF36
MBSRQ	0.417 (0.003)		
PCS SF36	0.388 (0.005)	0.539 (0.000)	
MCS SF36	0.355 (0.011)	0.334 (0.018)	0.298 (0.035)

Spearman correlation coefficient data are provided (*p* value).

### Factors associated with quality of life (SF-36), body image and self-esteem

**Figure 4:**
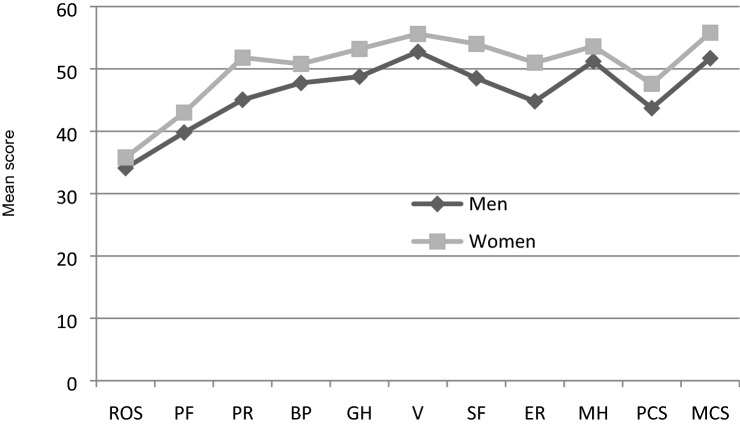
Rosenberg questionnaire, SF36 questionnaire (mean ± SD).

The influence of demographic variables on QoL, BI and SE in the participants living with amputations was assessed. For all the factors assessed, women had a higher level (Fig. 4) on average, although the difference between groups was not statistically significant in any of the cases. In terms of age, noteworthy was the “fitness-oriented behaviours” factor of the MBSRQ Body Image Questionnaire, which was significantly related to age, that is, the older the patient, the lower the score in this factor. Spearman’s correlation coefficient was − 0.477 (*p* value = 0.016).

## Discussion

The results of our study, related to the QoL analysis with the SF-36 questionnaire, highlight that people living with amputations have significantly lower mean levels than the comparison group in all subscales: PF, PR, BP, GH, V, SF, ER and MH.

QoL decreases in people with various musculoskeletal conditions as evidenced by the subscales, representing changes in physical condition^37^.

Supporting these results, in our study the subscale with the greatest difference is the one assessing PF.

They are also lower in the physical component summary (PCS) and in the mental component summary (MCS), although the difference is not statistically significant in the latter. These data are comparable with other studies^38^ that claim that QoL is lower than in the non-amputee population, and is not influenced by demographic, clinical or social factors^39^.

The BI results show that people living with amputations have a significantly lower mean score in the MBSRQ body image questionnaire compared to the comparison group, in agreement with other previously published studies^40,41^.

In terms of subscales, people with amputations also have significantly lower average levels in SIC, FOB, SAPA and COEA.

There are studies that have found a positive correlation between BI and physical activity using the MBSRQ^42^, indicating that lower limb amputees who regularly carry out physical activities have a higher BI than those who are not physically active.

The self-identity changes after a lower limb amputation go beyond the patient’s body image and functioning, affecting the patient’s awareness of the impairment and any future evolution^43^. We believe, like Mayer et al., that the use of a prosthesis helps to maintain a body schema in people living with amputations, as they sense the prosthetic limb in a similar manner to their intact leg, since, although they can see the prosthesis and are aware of the phantom limb, with the passing of time, their body, on walking, is not aware of the loss^40^.

SE is a reflection of a person’s value. In our study, there were no significant differences on Rosenberg’s Self-Esteem scale between people living with amputations and the comparison group, although the latter scored slightly higher.

Holzer et al. showed that lower limb amputation does not reflect significant differences in SE with respect to the comparison group^41^, thus coinciding with our results.

The strength of our study resides in the fact there is no national registry of this type of patients. All available patients have been included (no selection bias is recognised), precisely to discover demographic characteristics, and evaluate their situation in terms of quality of life, in order to be able to take measures and improve their medical care situation.

Although the optimal sample size has been reached, which is similar to other studies, such as Quigley’s study with a transtibial amputation cohort of 23 patients^7^, the sample size is considered a limitation, and having a larger sample would allow us to confirm our results.

## Conclusion

By way of conclusion, this study shows that lower limb amputations significantly influence QoL, both in terms of PCS and in MCS, and in all subscales (PF, PR, BP, GH, V, SF, ER, MH).

The results also show that amputation has a significant influence on the BI of patients with unilateral transtibial amputations, and this is reflected in subscales, SIC, FOB, SAPA and COEA.

SE seems to be an aspect that is not significantly affected by lower limb amputation, although people living with amputations had a lower mean score on the RSE scale compared to the control group.

We consider it is highly relevant to assess QoL, BI and SE in patients after lower limb amputation.

In an amputation process, psychological intervention is of paramount importance, since it contributes to generating an adaptation to the new life. In addition, there is greater adherence to treatment, leading to a successful recovery and a restructured life project, notably improving the quality of life.

Since aesthetics and the perception of beauty have a great impact on our society, and bearing in mind that the main pillars of the perception of one’s appearance are body image and self-esteem, we try to provide relevant data in this study to help in the treatment and adaptation of the person living with an amputation to his/her new situation. Our contribution could be useful to clinicians and researchers, helping to improve outcomes for people living with amputations.
